# H5N1 Influenza A Virus PB1-F2 Relieves HAX-1-Mediated Restriction of Avian Virus Polymerase PA in Human Lung Cells

**DOI:** 10.1128/JVI.00425-18

**Published:** 2018-05-14

**Authors:** B. Mazel-Sanchez, I. Boal-Carvalho, F. Silva, R. Dijkman, M. Schmolke

**Affiliations:** a Department of Microbiology and Molecular Medicine, CMU, University of Geneva, Geneva, Switzerland; b Institute of Virology and Immunology, Bern & Mittelhausern, Switzerland; c Department of Infectious Diseases and Pathobiology, Vetsuisse Faculty, University of Bern, Bern, Switzerland; St. Jude Children's Research Hospital

**Keywords:** H5N1, HAX-1, influenza A virus, PA, PB1-F2, polymerases, zoonosis

## Abstract

Highly pathogenic influenza A viruses (IAV) from avian hosts were first reported to directly infect humans 20 years ago. However, such infections are rare events, and our understanding of factors promoting or restricting zoonotic transmission is still limited. One accessory protein of IAV, PB1-F2, was associated with pathogenicity of pandemic and zoonotic IAV. This short (90-amino-acid) peptide does not harbor an enzymatic function. We thus identified host factors interacting with H5N1 PB1-F2, which could explain its importance for virulence. PB1-F2 binds to HCLS1-associated protein X1 (HAX-1), a recently identified host restriction factor of the PA subunit of IAV polymerase complexes. We demonstrate that the PA of a mammal-adapted H1N1 IAV is resistant to HAX-1 imposed restriction, while the PA of an avian-origin H5N1 IAV remains sensitive. We also showed HAX-1 sensitivity for PAs of A/Brevig Mission/1/1918 (H1N1) and A/Shanghai/1/2013 (H7N9), two avian-origin zoonotic IAV. Inhibition of H5N1 polymerase by HAX-1 can be alleviated by its PB1-F2 through direct competition. Accordingly, replication of PB1-F2-deficient H5N1 IAV is attenuated in the presence of large amounts of HAX-1. Mammal-adapted H1N1 and H3N2 viruses do not display this dependence on PB1-F2 for efficient replication in the presence of HAX-1. We propose that PB1-F2 plays a key role in zoonotic transmission of avian H5N1 IAV into humans.

**IMPORTANCE** Aquatic and shore birds are the natural reservoir of influenza A viruses from which the virus can jump into a variety of bird and mammal host species, including humans. H5N1 influenza viruses are a good model for this process. They pose an ongoing threat to human and animal health due to their high mortality rates. However, it is currently unclear what restricts these interspecies jumps on the host side or what promotes them on the virus side. Here we show that a short viral peptide, PB1-F2, helps H5N1 bird influenza viruses to overcome a human restriction factor of the viral polymerase complex HAX-1. Interestingly, we found that human influenza A virus polymerase complexes are already adapted to HAX-1 and do not require this function of PB1-F2. We thus propose that a functional full-length PB1-F2 supports direct transmission of bird viruses into humans.

## INTRODUCTION

Highly pathogenic avian influenza A viruses (IAV) pose a continuing threat to human and animal health and bear the potential for new influenza pandemics. Since 2003, 860 confirmed cases of human H5N1 IAV have been reported to the WHO (1), with an estimated mortality rate of about 50%. In the same time frame, millions of domestic birds were culled or died as a consequence of H5N1 IAV infection, resulting in a substantial economic burden (2).

Avian influenza viruses face tremendous challenges when overcoming the species barrier to replicate in mammalian hosts. In fact, aside from limited family cluster infections (3), there are currently no examples of continuous human-to-human transmission for the H5N1 subtype, despite a very high capacity of the virus to acquire adaptive mutations. The reasons for this threshold of adaptation to the human hosts can be interpreted as a poor adjustment to proviral host factors, an increased sensitivity to host restriction factors, or a combination of the two. The viral RNA-dependent RNA polymerase complex of avian IAV seems to require a number of host-specific adaptations to function fully in mammalian cells. It is largely unclear which factors define a successful zoonotic avian IAV. One viral protein which has repeatedly been proposed to contribute to pathogenicity and lethality of pandemic and zoonotic IAV is PB1-F2 (4–8). This short viral peptide is encoded from a +1 open reading frame (ORF) of segment 2. Full-length PB1-F2 is predominantly found in viruses of avian origin (9), suggesting that its natural function evolved in these host species. Consequently, full-length PB1-F2 ORFs were found in all pandemic viruses of avian origin and in zoonotic, highly pathogenic avian IAV. The presence of a full-length PB1-F2 and certain sequence variations in its C terminus has been associated with increased viral pathogenicity and immune modulation (10, 11). However, depending on the investigated strain and the cellular context, PB1-F2 can either promote or suppress antiviral signaling (11, 12). In human hosts, H1N1 IAV descending from the 1918 Spanish influenza virus have the full-length PB1-F2 ORF; however, from the late 1940s, it becomes truncated after 59 amino acids (aa) (9). H1N1 IAV descending from the 2009 swine flu IAV carry an even shorter PB1-F2 due to a premature STOP codon (13). Among human H3N2 strains, a comparative study showed effects of PB1-F2 on viral pathology only in isolates recovered directly after the introduction into the human population, while in later isolates PB1-F2 seems to play only a minor role due to mutations or truncations (5, 10, 14). Taken together, these findings imply that PB1-F2 could have an impact on the human host in viruses recently derived from avian hosts, e.g., during a direct zoonotic infection of humans with avian H5N1 strains.

Intriguingly, PB1-F2 does not contain an intrinsic enzymatic function and thus likely depends on recruitment of host factors to evoke a phenotype during virus infection. In this study, we identified novel host proteins interacting with PB1-F2 of a zoonotic H5N1 IAV in the context of viral infection. We found that PB1-F2 binds to and colocalizes with HCLS-1-associated protein 1 (HAX-1), a poorly characterized restriction factor of IAV polymerase function which binds to the PA subunit (15). Interestingly, HAX-1 robustly restricts H5N1 polymerase function by targeting PA, while H1N1 PA promotes resistance to HAX-1-dependent inhibition. We demonstrate that PB1-F2 of H5N1 IAV balances the HAX-1-mediated restriction of PA by direct competition, thus allowing these zoonotic viruses to successfully replicate in human hosts.

## RESULTS

### Identification of host interaction partners of PB1-F2.

**FIG 1:**
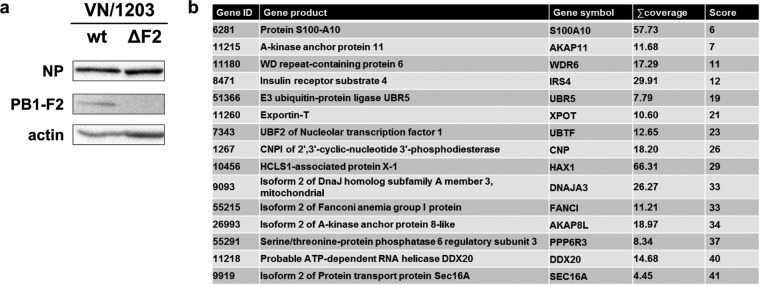
Identification of PB1-F2 interactors. (a) Western blot showing the efficacy of PB1-F2 deletion in VN/1203. MDCK cells were infected at an MOI of 5 and lysed 24 h postinfection. Lysates were processed through Western blotting for detection of the cellular protein actin and the viral proteins NP (nucleoprotein) and PB1-F2. (b) Table showing the top 15 interactors identified by mass spectrometry.

Full-length PB1-F2 is predominantly maintained in avian isolates. Mutations and/or truncations occur in the process of adaptation to mammalian hosts. In order to test the role of PB1-F2 at the human-bird interface, we decided to use PB1-F2 from a human H5N1 isolate (A/Viet Nam/1203/2004 [VN/1203]). This highly pathogenic avian-origin virus was isolated from a human patient after infection from an avian host (16, 17) and thus provides a good model to study the function of accessory viral proteins involved in overcoming species barriers. To avoid the inconvenience of biosafety level 3 (BSL3) lab work, we chose a low-pathogenicity mutant lacking the multibasic cleavage site (18). We first generated a PB1-F2-deficient virus by taking advantage of reverse genetics, applying a strategy proposed by Tauber and colleagues (19). We introduced two stop codons (see Materials and Methods) that abolish expression of PB1-F2 (VN/1203 ΔF2) (Fig. 1a) without affecting the amino acid sequence encoded by the PB1 ORF. In order to identify host proteins binding to PB1-F2 during infection, we reconstituted this virus, VN/1203 ΔF2, by infecting HEK 293T cells (293T cells) expressing N-terminally Flag-tagged PB1-F2 of VN/1203. Proteins interacting with PB1-F2 were identified after Flag-specific pulldown and mass spectrometry (Fig. 1b; see also Tables S1 and S2 in the supplemental material).

We found HCLS1-associated protein X-1 (HAX-1) among the top 10 host proteins interacting with VN/1203 PB1-F2. This protein was recently described to bind to influenza A virus (A/WSN/1933) PA and prevent it from entering the nucleus, thus reducing viral transcription and replication (15). We postulated that PB1-F2 of avian IAV might counteract this inhibition and decided to study the interaction of HAX-1 and PB1-F2 in more detail.

### The PB1-F2 C terminus interacts with HAX-1.

**FIG 2:**
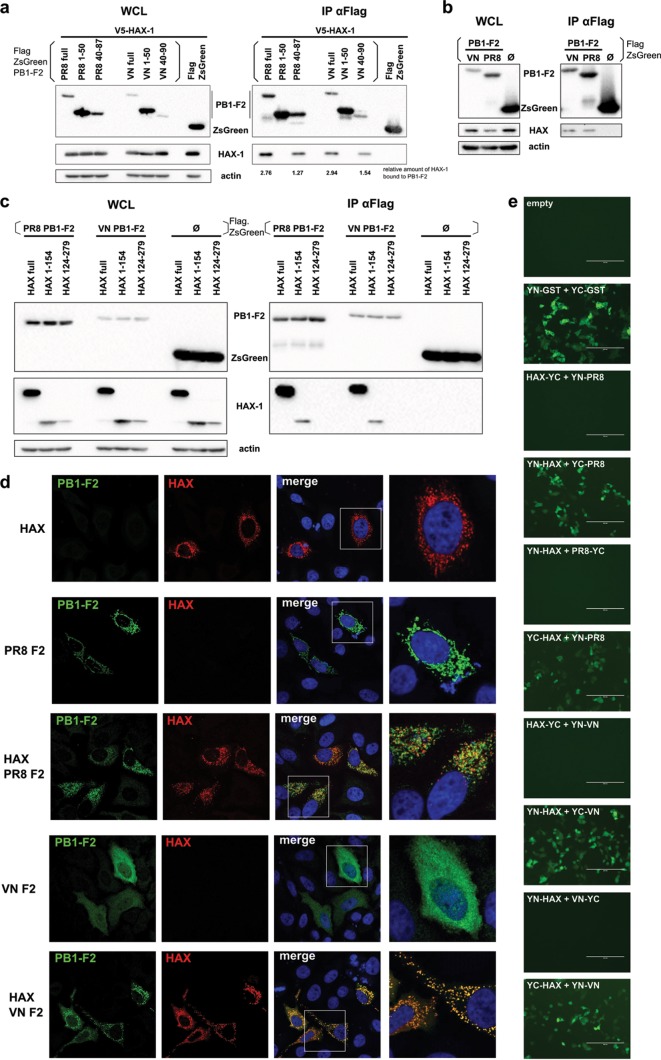
PB1-F2 and HAX-2 interact in coimmunoprecipitation and colocalize in IF. (a to c) 293T cells were transfected with each indicated protein cloned into the mammalian expression plasmid pCAGGS. Whole-cell lysates (WCLs) were collected 24 h later and processed through regular IP (b) or sequential IP (a and c) using a monoclonal antibody against Flag-tagged proteins. Samples were then analyzed by Western blotting. (d) HeLa cells were transfected with V5-HAX-1 and Flag-PB1-F2 proteins. Cells were fixed at 48 h, processed for detection of HAX-1 (red) and PB1-F2 (green) by immunofluorescence, and stained with DAPI. (e) YN and YC fragments of YFP were cloned at the N terminus or C terminus of glutathione *S*-transferase (GST; self-interacting control), HAX-1, and VN.PB1-F2. HeLa cells were transfected with indicated combinations of those constructs and incubated for 40 h at 37°C and then 4 h at 33°C. Images were taken using the EVOS cell imaging system.

In order to gain insight into the interaction of PB1-F2 with HAX-1, we first confirmed the mass spectrometry data in independent immunoprecipitation (IP) experiments. We tested PB1-F2 of VN/1203 and of H1N1 A/Puerto Rico/8/1934 (PR/8) and showed that they both efficiently pulled down HAX-1 (Fig. 2a).

**FIG 3:**
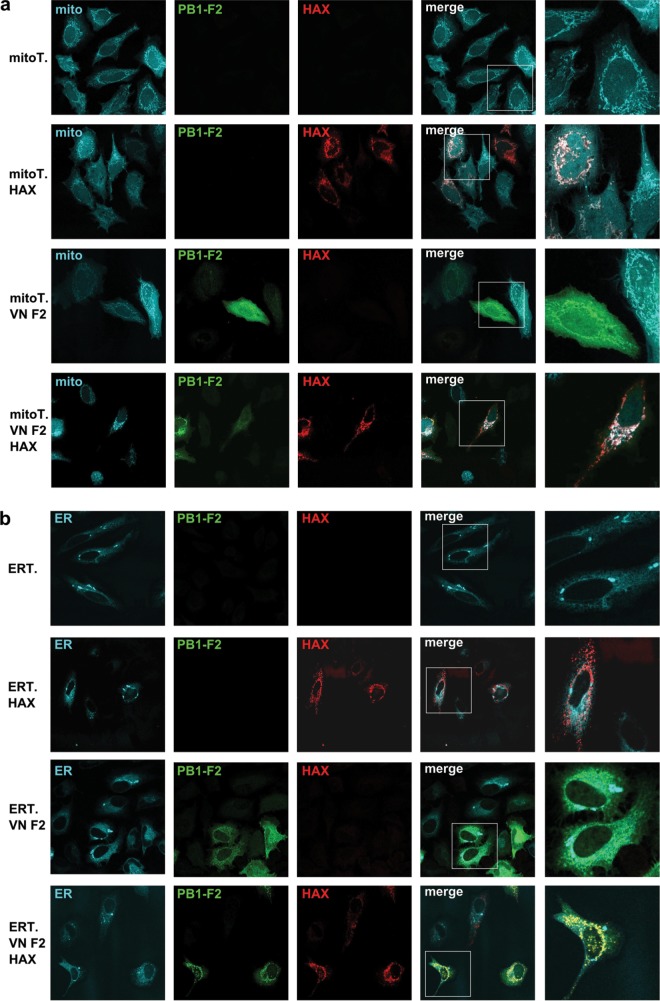
PB1-F2 interacts with HAX-1 at the level of the mitochondria. (a and b) HeLa cells were transfected with V5-HAX-1 and Flag-PB1-F2 proteins with an mTurquoise fluorescent protein addressed either to the mitochondria (mitoT.) (a) or to the endoplasmic reticulum (ERT.) (b). Cells were fixed at 48 h and processed for detection of HAX-1 (red) and PB1-F2 (green) by immunofluorescence.

We then tested which portion of PB1-F2 binds to HAX-1 and constructed a C-terminally truncated version (aa 1 to 50) and an N-terminally truncated version (aa 40 to 90) of PB1-F2. Only the N-terminally truncated PB1-F2 construct pulled down HAX-1 efficiently, locating the interaction domain to the C-terminal region of PB1-F2 (Fig. 2a). It is noteworthy that PB1-F2 consistently pulled down endogenous HAX-1 (Fig. 2b). However, we could not determine with confidence the domain for interaction of HAX-1 with full-length PB1-F2. Both the N-terminal portion and the C-terminal portion of HAX-1 were less stably expressed than the full length, and only the N-terminal part pulled down with PB1-F2. Of note, the C-terminal portion was even less stable, which might explain its efficient pulldown (Fig. 2c). By confocal immunofluorescence (IF), we confirmed that VN/1203 and PR/8 PB1-F2 colocalize with HAX-1. Interestingly, VN/1203 PB1-F2 diffuses in the cytoplasm when expressed alone, while it focuses to punctate structures in the presence of HAX-1, supporting an interaction of PB1-F2 with HAX-1 (Fig. 2d). Meanwhile, PR/8 PB1-F2 localizes to punctate structures independently of HAX-1. The HAX-1 protein was described to localize predominantly to mitochondria and partially to the endoplasmic reticulum (ER). Using fluorescent proteins addressed either to the mitochondria or to the ER, we confirmed that PB1-F2 of VN/1203 and HAX-1 colocalize at the mitochondria but not at the ER (Fig. 3). Finally, we tested the interaction of PB1-F2 with HAX-1 in a bimolecular fluorescence complementation (BiFC) system, where proximal proteins coupled to one portion of a split yellow fluorescent protein (YFP) result in formation of an excitable fluorophore (20). Interaction of YN-PB1-F2 with YC-HAX-1 and YC-PB1-F2 with YN-HAX resulted in a strong YFP signal, indicating close proximity of both proteins (Fig. 2e). Of note, we could not reproduce the mitochondrial localization of this interaction in this assay, likely because PB1-F2 is as big as the split-YFP tag, which might cause subcellular misallocation.

### PB1-F2-deficient viruses are sensitive to inhibition by HAX-1.

**FIG 4:**
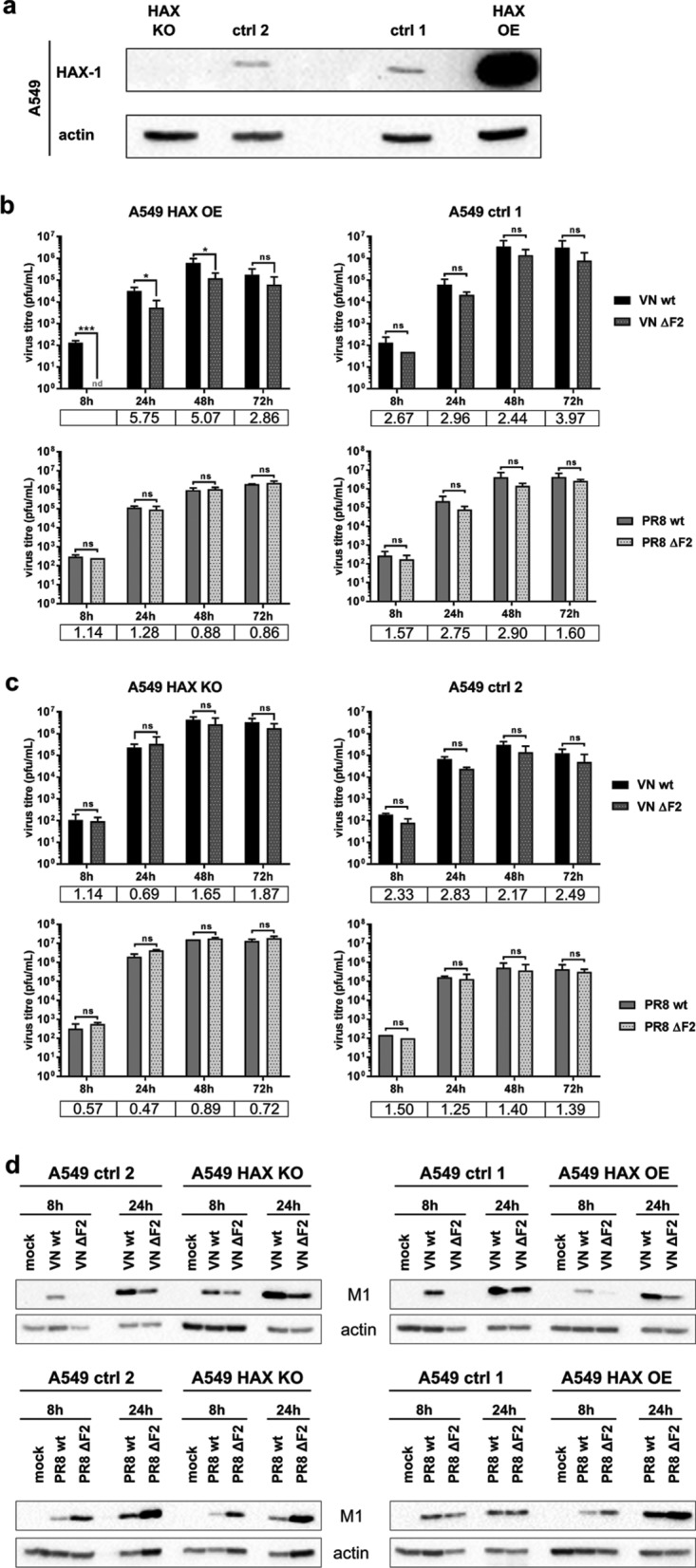
HAX-1 inhibits virus replication in a PB1-F2-dependent manner. (a) Western blot showing the efficiency of HAX-1 knockout and HAX-1 overexpression in the cells used for the growth curve. (b and c) Different A549 cell lines were infected at an MOI of 0.01 with either VN/1203 wt, VN/1203 ΔF2, PR/8 wt, or PR/8 ΔF2 virus with TPCK-treated trypsin at 0.1 μg/ml. Supernatants were collected at the indicated time point postinfection, and virus titer was assessed by plaque assay on MDCK cells. Bars represent means (*n* = 3), with error bars indicating SDs. Fold changes between wt and ΔF2 viruses are indicated in the table below each graph. Student *t* test was used to compare virus titer at each time point. n.s., nonsignificant; n.d., not detected. *, *P* < 0.05; ***, *P* < 0.001. (d) Different A549 cell lines were infected at an MOI of 1 with either VN/1203 wt, VN/1203 ΔF2, PR/8 wt, or PR/8 ΔF2 virus. At the indicated time point postinfection, cells were lysed and processed through WB.

We postulated that PB1-F2 could be required to overcome HAX-1-dependent inhibition of the viral polymerase complex. Hsu and colleagues recently showed a 2-fold reduction of viral titers of A/WSN/1933 H1N1 in control H1299 lung epithelial cells compared to HAX-1 short hairpin RNA (shRNA) knockdown H1299 cells (15). We decided to use a clustered regularly interspaced short palindromic repeat (CRISPR)/Cas9 knockout (KO) approach to fully remove endogenous HAX-1 from A549 lung epithelial cells and complemented this approach with a HAX-1-overexpressing cell line (Fig. 4a). We could thus test the sensitivity of PB1-F2-expressing and PB1-F2-deficient viruses in the presence of various levels of HAX-1. In the presence of high levels of HAX-1, we found reduced replication only of the zoonotic H5N1 strain. This reduction was more pronounced in the virus lacking PB1-F2 (VN/1203 ΔF2) than in the wild-type (wt) virus. In contrast, replication of the H1N1 mammalian IAV strain did not appear to depend on PB1-F2 and replicated efficiently in cells expressing high levels of HAX-1 (Fig. 4b). In cells lacking HAX-1 expression, we could observe a general increase of viral titer independent of the virus strain or the presence/absence of PB1-F2 (Fig. 4c). Of note, we observed consistently a reduction of viral titers for VN/1203 ΔF2 compared to wt virus in control cells. However, this reduction did not reach statistical significance. To further demonstrate that endogenous levels of HAX-1 already impact the PB1-F2-deficient H5N1 virus, we compared viral protein expression in A549 wt cells. After 8 h and 24 h, VN/1203 ΔF2-infected A549 wt cells contained lower levels of viral matrix protein M1 than did VN/1203 wt-infected cells (Fig. 4d). In accordance with the viral growth curves, there was no difference in M1 levels of VN/1203 wt- and VN/1203 ΔF2-infected HAX-1-deficient cells. For PR/8, we rather found increased levels of M1 in case of VN/1203 ΔF2, a phenomenon that does not correlate with the viral growth curves and currently lacks explanation.

Taken together, these data suggest that HAX-1 indeed poses a specific barrier to zoonotic avian viruses for replication in human cells and that PB1-F2 is required to overcome this barrier.

### Inhibition of virus replication does not correlate with apoptosis.

**FIG 5:**
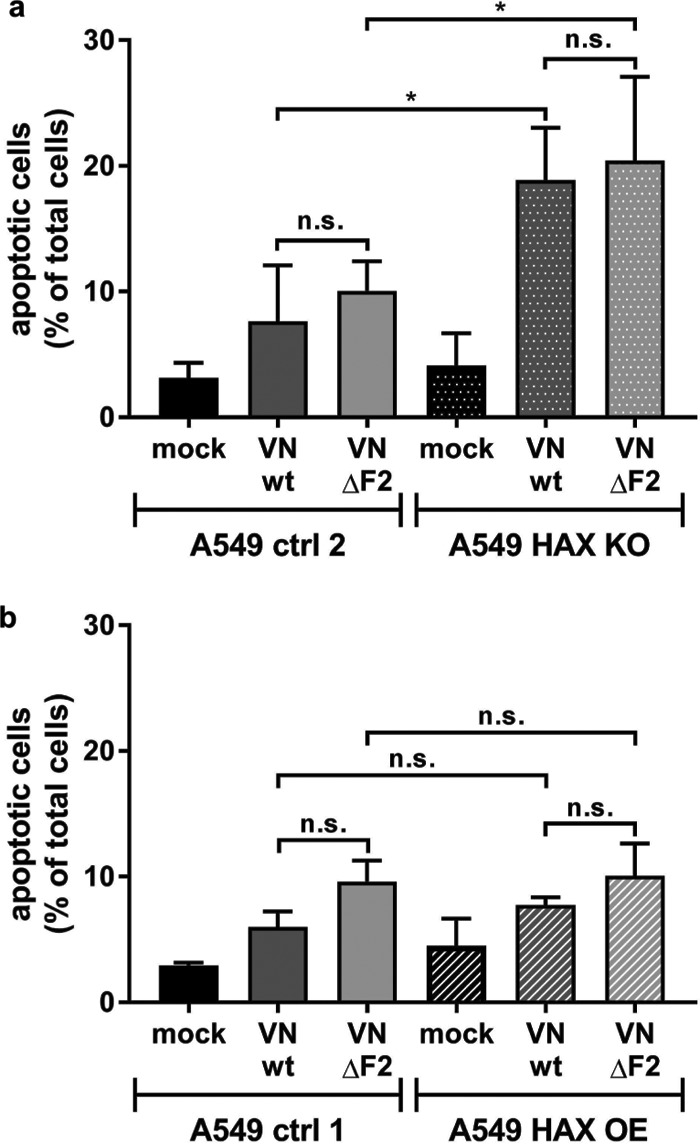
HAX-1 overexpression inhibits viral replication in an apoptosis-independent manner. (a and b) Different A549 cell lines were infected at an MOI of 5 for 24 h, collected, and stained for annexin V and Zombie Red. The percentages of total apoptotic cells (annexin V positive, Zombie positive, and double positive) are plotted. Values represent means (*n* = 3) with SDs. One-way analysis of variance (ANOVA) was used to compare samples. *, *P* < 0.05.

HAX-1 was shown to have antiapoptotic functions (21); thus, we tested if IAV infection induces different levels of apoptosis in HAX-1 ctrl and HAX-1 KO A549 cells (Fig. 5a). In contrast to the study by Hsu et al. (15), all viruses induced more apoptosis in HAX-1-deficient cells, with no significant difference between wt and ΔF2 variants. We speculate that increased apoptosis might enhance release of viral ribonucleoprotein complexes, contributing to the overall increased virus titers in HAX-1 KO cells, as demonstrated before (22). In A549 cells overexpressing HAX-1, we did not observe reduced apoptosis compared to that in A549 wt cells (Fig. 5b). This rules out an impact of apoptotic cell death on reduced virus replication in cells expressing high levels of HAX-1.

### HAX-1 inhibits viral polymerase of avian viruses.

**FIG 6:**
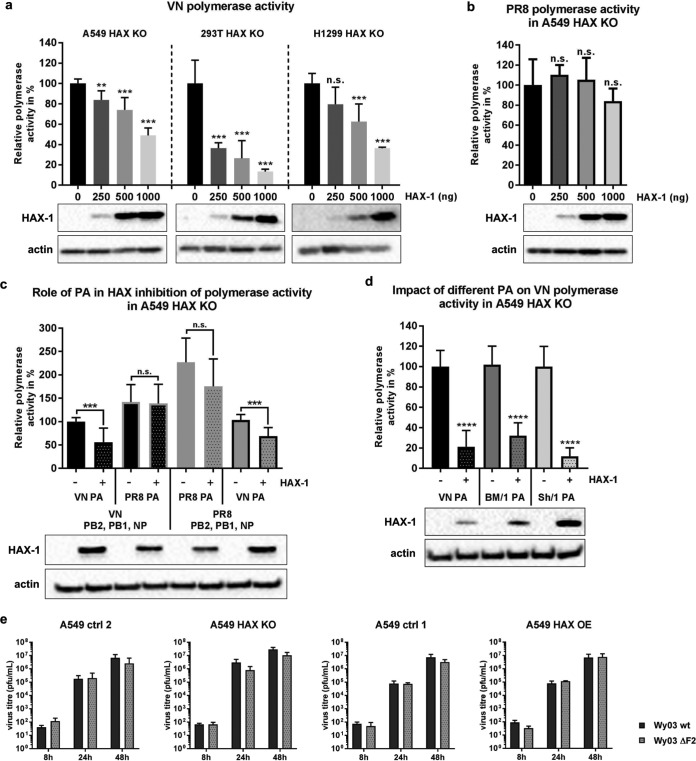
HAX-1 inhibits viral polymerase activity in a PA-dependent manner. (a to d) Mini-replicon assays were performed in A549, H1299, and 293T cells lacking HAX-1 expression. Cells were transfected with a plasmid carrying a firefly luciferase-based minigenome and one encoding Renilla luciferase under the control of a constitutive promoter, as well as the following plasmids: 10 ng of pCAGGS.VN-PB2, 10 ng of pCAGGS.VN-PB1, 10 ng of pCAGGS.VN-PA, 20 ng of pCAGGS.VN-NP, and different amounts of pCAGGS.HAX-1 (a); 10 ng of pCAGGS.PR8-PB2, 10 ng of pCAGGS.PR8-PB1, 10 ng of pCAGGS.PR8-PA, 20 ng of pCAGGS.PR8-NP, and different amounts of pCAGGS.HAX-1 (b); either 10 ng of pCAGGS.VN-PB2, 10 ng of pCAGGS.VN-PB1, 20 ng of pCAGGS.VN-NP, or 10 ng of pCAGGS.PR8-PB2, 10 ng of pCAGGS.PR8-PB1, and 20 ng of pCAGGS.PR8-NP with the 10-ng PA segment of either pCAGGS.VN-PA or pCAGGS.PR8-PA and with or without 500 ng of pCAGGS.HAX-1 (c); and 10 ng of pCAGGS.VN-PB2, 10 ng of pCAGGS.VN-PB1, and 20 ng of pCAGGS.VN-NP with the 10-ng PA segment of pCAGGS.VN-PA or pCAGGS.BM/1-PA or pCAGGS.Sh/1-PA (d). Cells were lysed 24 h posttransfection, and lysates were subjected to luciferase assay. Values represent means from triplicate experiments, with SDs. One-way ANOVA was used. **, *P* < 0.01; ***, *P* < 0.001; ****, *P* < 0.0001. Cell lysates were also used for WB to visualize HAX-1 expression and actin. (e) Different A549 cell lines were infected at an MOI of 0.01 with either Wy/03 wt or Wy/03 ΔF2 virus with TPCK-treated trypsin at 0.1 μg/ml. Supernatants were collected at the indicated time point postinfection, and virus titer was assessed by plaque assay on MDCK cells. Bars represent means (*n* = 3), with SDs.

We used a mini-replicon system to corroborate the specific inhibitory effect of HAX-1 on avian IAV polymerase complexes. A549 HAX-1 KO cells were transfected with increasing amounts of expression plasmids for HAX-1 as well as the three subunits of the IAV polymerase, the IAV nucleoprotein, and a luciferase reporter under the control of the IAV promoter sequence. In the case of H5N1 polymerase, we observed a significant and dose-dependent reduction of viral polymerase-driven reporter gene production. The same effect was observed in HAX-1-deficient 293T and H1299 cells complemented with different doses of exogenous HAX-1 (Fig. 6a). In contrast, the H1N1 polymerase function was not affected (Fig. 6b). To demonstrate that PA and not another subunit of the polymerase complex is the target of HAX-1-mediated inhibition, we replaced the H5N1 PA subunit with the H1N1 PA subunit while maintaining the residual components of the H5N1 mini-replicon system. We observed that the H5N1 polymerase sensitivity to HAX-1 was overcome by introduction of an H1N1 PA subunit (Fig. 6c). This result shows that H5N1 PA is the target of HAX-1 function. In agreement with this, replacement of H1N1 PA by H5N1 PA in the H1N1 mini-replicon renders the polymerase complex susceptible to HAX-1 inhibition (Fig. 6c, right). Next we tested the sensitivity of PAs from two additional avian-origin viruses which were shown to directly infect humans (A/Brevig Mission/1/1918 H1N1 and A/Shanghai/1/2013 H7N9). The two PAs showed equal sensitivities to HAX-1 in the context of the VN polymerase complex (Fig. 6d). Finally, we tested a recent human H3N2 strain for its sensitivity to HAX-1. In contrast to prepandemic and current human H1N1 strains, human H3N2 viruses carry a full-length PB1-F2 ORF. The PA subunit of this virus was incompatible with the H5N1 mini-replicon system and yielded only background luciferase signals, even in the absence of HAX-1 (data not shown). We thus decided to rescue a PB1-F2-deficient mutant of this virus and compare it to the parental strain. Deletion of PB1-F2 does not affect replication of H3N2 IAV independently of the HAX-1 levels of the host cell (Fig. 6e). From these results, we conclude that PA subunits of zoonotic viruses are sensitive to HAX-1-dependent restriction, while human-adapted viruses are insensitive and do not require PB1-F2 to counteract HAX-1.

### HAX-1 interacts with the PA subunit of the IAV polymerase.

**FIG 7:**
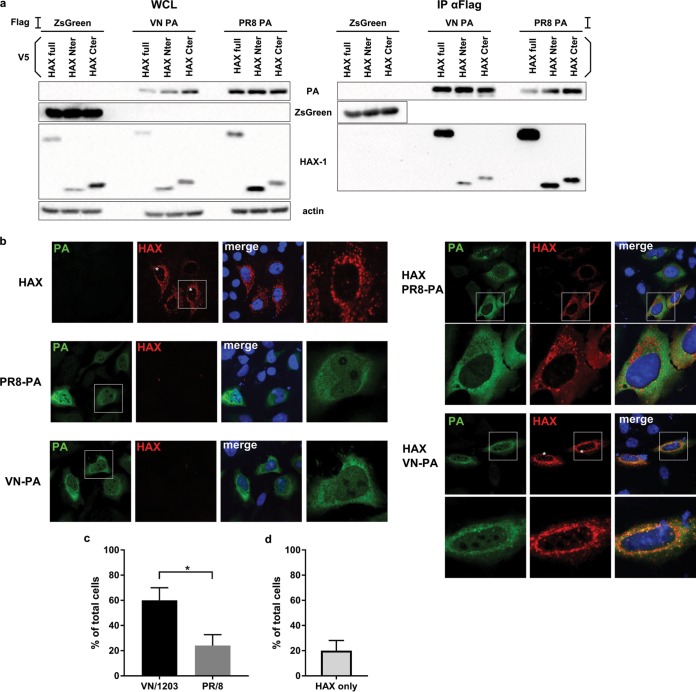
HAX-1 interacts with the PA subunit of the polymerase. (a) 293T cells were transfected with each indicated protein cloned into the mammalian expression plasmid pCAGGS.V5-HAX-1 and pCAGGS.Flag-VN-PA. Cell lysates were collected 48 h later and processed through immunoprecipitation using a monoclonal antibody against Flag-tagged proteins. Samples were then analyzed by Western blotting. (b) HeLa cells were transfected with V5-HAX-1 and Flag-PA proteins from either VN/1203 or PR/8. Cells were fixed at 48 h and processed for detection of HAX-1 (red) and PA (green) by immunofluorescence and stained with DAPI. An asterisk indicates cells where HAX-1 is present in the cytoplasm. (c and d) Graphs represent the percentages of cells where HAX-1 localizes to the nucleus. Bars represent the means, with SEMs (*n* = 25). (c) In samples transfected with both HAX-1 and PA, we took into account cells that were doubly positive. *, *P* < 0.05. (d) In samples transfected with HAX-1 only, we took into account every HAX-1-positive cells.

The main role of PA for viral replication is providing capped primers for viral mRNA synthesis via endonucleolytic cap snatching (23). This process initiates viral transcription in the nucleus. Nevertheless, PA was also found to localize to the cytoplasm, probably independently of the other components of the viral polymerase complex (24), with yet unclear function. To study the interaction of PA with HAX-1 in a more detailed manner, we first performed pulldown experiments. Surprisingly, both H1N1 and H5N1 PA interact with HAX-1 (Fig. 7a).

Then we checked in which subcellular compartment H1N1 and H5N1 PAs interact with HAX-1. We transfected HeLa cells with the following proteins alone or in combination: HAX-1, VN/1203 PA, and PR/8 PA (Fig. 7b). We observed that both the PAs of VN/1203 and PR/8 colocalize with HAX-1 in the cytoplasm. Interestingly, we found that HAX-1 also colocalizes with H5N1 PA in nuclear foci in about 60% of doubly transfected cells, while HAX-1 is present in nuclear foci in only 20% of cells doubly transfected with H1N1 PA and HAX-1 (Fig. 7c). When expressed alone, HAX-1 predominantly localizes to the mitochondria but can be found in the nuclei of some cells (about 20%) (Fig. 7d).

### PB1-F2 competes directly with PA for HAX-1 binding.

**FIG 8:**
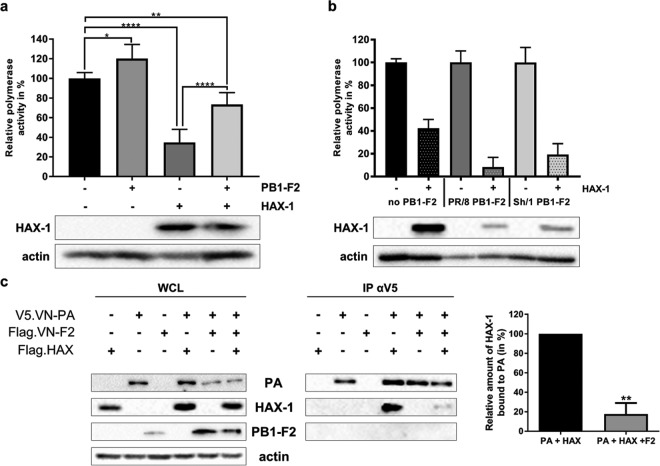
PB1-F2 relieves HAX-1 inhibition of PA by competing out their interaction. (a and b) Mini-replicon assay was performed in A549 HAX KO cells. Subconfluent cells were transfected with a plasmid carrying a firefly luciferase-based minigenome and one encoding Renilla luciferase under the control of a constitutive promoter, as well as the following plasmids: 40 ng of pCAGGS.VN-PB2, 40 ng of pCAGGS.VN-PB1, 40 ng of pCAGGS.VN-PA, 80 ng of pCAGGS.VN-NP, and, when indicated, 500 ng of pCAGGS.VN-PB1-F2 and 250 ng of pCAGGS.HAX-1 (a) and 40 ng of pCAGGS.VN-PB2, 40 ng of pCAGGS.VN-PB1, 40 ng of pCAGGS.VN-PA, 80 ng of pCAGGS.VN-NP, and, when indicated, 250 ng of pCAGGS.HAX-1 and PB1-F2 from different virus strains, pCAGGS.PR8-PB1-F2, or pCAGGS.Sh/1-PB1-F2 (b). Cells were lysed 24 h after transfection, and lysates were subjected to luciferase assay. Values represent means from duplicate experiments, with SDs. One-way ANOVA was used. *, *P* < 0.1; **, *P* < 0.01; ****, *P* < 0.0001. Cell lysates were also used for WB to visualize HAX-1 expression and actin. (c) 293T cells were transfected with each indicated protein cloned into the mammalian expression plasmid pCAGGS. Cell lysates were collected 48 h later and processed through immunoprecipitation using a monoclonal antibody against Flag-tagged proteins. Samples were then analyzed by Western blotting. The Western blot shown is representative of three experiments. The graph represents the relative amounts of HAX-1 bound to PA in the presence or absence of PB1-F2. Means were compared with Student *t* test. **, *P* < 0.01 (*n* = 3).

Finally, we aimed at understanding mechanistically how PB1-F2 overcomes the restriction of PA by HAX-1 in H5N1-infected cells. Using the same mini-replicon approach as described above, we could show that PB1-F2 partially restores polymerase function when coexpressed with HAX-1 (Fig. 8a). Expression of PB1-F2s from PR/8 (H1N1) and the zoonotic H7N9 A/Shanghai/1/2013 did not restore polymerase function of H5N1 polymerase in the presence of HAX-1 (Fig. 8b). Mechanistically, the simplest model would describe a direct competition of H5N1 PB1-F2 and PA for HAX-1 binding. In order to support this model, we overexpressed all three proteins and pulled down PA. We demonstrated that VN/1203 PA interacts with HAX-1 and that this interaction is diminished by about 80% in the presence of VN/1203 PB1-F2 (Fig. 8c; average from three independent pulldown experiments). This result suggests that PB1-F2 replaces PA at the HAX-1 binding site, thus allowing the release of the polymerase subunit from HAX-1 inhibition.

## DISCUSSION

IAV is a highly adaptive organism, capable of adjusting to a diverse group of host organisms (25). Adaptation to new host species requires adjustments of virus host interfaces on potentially each level of viral replication. Consequently, zoonotic transmission of IAV between very distantly related hosts, such as domestic bird species and humans, requires substantial mutagenesis (reviewed in reference 26). At the level of the viral polymerase complex the differences in host adaptation become especially obvious (27). Avian IAV polymerase complexes tend to show reduced functionality in human cells and vice versa (28). Adaptive mutations in all three subunits and nucleoprotein (NP) of avian IAV polymerase complexes were identified (27–32). Mehle and colleagues showed that replacement of an avian virus PA with a human virus PA gives a critical replication advantage for avian virus in mammalian cells (32). The driving forces behind most of these changes are likely incompatibilities with required host factors or specific restriction factors. Species-specific sensitivity of viral polymerase complexes was previously described for myxovirus resistance A (MxA), probably the most potent mammalian interferon-stimulated gene (ISG) product acting predominantly against poorly adapted avian IAVs (33). In contrast to MxA, HAX-1 is not a classical ISG product but rather is constantly expressed at a fairly high level in a wide range of tissues, including lungs (34). HAX-1 was extensively studied in the context of calcium signaling and was shown to prevent ER stress-induced apoptosis (21; reviewed in references 35 and 36). Interestingly, a number of proteins encoded by viruses other than IAV were shown to interact with HAX-1 and were implicated in modulating apoptotic host responses in this way (37–39).

HAX-1 was previously described to antagonize the PA subunit of IAV (15). Although that study used A/WSN/1933, a mouse-adapted H1N1 strain, the authors observed a subtle (2-fold) reduction in viral replication in the presence of endogenous levels of HAX-1 compared to HAX-1 knockdown H1299 cells. We confirmed these findings using a clean knockout approach; however, in contrast to the initial study, we found a substantial increase in virus-induced apoptosis in HAX-1 knockout cells. This is in line with the antiapoptotic role described for HAX-1 but contradicts the study by Hsu et al. (15). Apoptosis can have a positive effect on release of viral RNPs from the nucleus, potentially by activation of host caspases and cleavage of nuclear pore complexes (22); this might explain the generally increased replication of both human and avian IAV in HAX-1 knockout cells. Consequently, we cannot rule out that in these cells apoptosis indeed plays a role in the enhanced replication of all viruses tested in this study, independent of their sensitivity to HAX-1.

Our data further indicate that the localization of HAX-1–PA interaction might be key to understanding its specificity. While H1N1 PA interacts with HAX-1 predominantly in the cytoplasm, avian H5N1 PA forms foci with HAX-1 in the nucleus, the compartment of viral transcription and replication. The nature of these foci requires further investigation. For H1N1, we postulate that a proportion of PA might retain HAX-1 in the cytoplasm, preventing it from interacting with the replication process in the nucleus, while H5N1 PA translocates to the nucleus with HAX-1. Sequence differences in PA of PR/8 or VN/1203 might explain this difference in viral sensitivity to HAX-1. A number of residues in avian polymerase subunit PA were identified to change upon viral adaptation to human host cells. Bussey and colleagues identified two residues in the N-terminal part of the PA of 2009 H1N1 PA that enhance viral polymerase activity in mammalian cells (30). This PA is of avian origin but likely adapted to a mammalian host due to the long prevalence in the swine host (40).

When trying to determine how certain viruses (such as zoonotic H5N1 strains) overcome this restriction, we found that PB1-F2, an accessory peptide highly prevalent in avian IAV, binds to HAX-1 and compensates the restriction of PA in the mini-replicon assay. H1N1 PB1-F2 did not rescue HAX-1-dependent suppression of PA. Surprisingly, the same was true for a second zoonotic virus, H7N9. It could be that zoonotic H7N9 viruses found alternative strategies to circumvent innate restriction of the polymerase complex through the mammalian host. Mazur and colleagues showed enhanced IAV polymerase function in the presence of PB1-F2, supposedly the result of interaction with free PB1 (41). This effect was later shown to be limited to the A/Puerto/Rico/8/1934 polymerase complex (42) and does not appear to play a role in the function of PB1-F2 proposed here.

Full-length PB1-F2 is expressed not only in pathogenic H5N1 and low-pathogenicity H7N9 isolates but also in reassortant pandemic IAV (e.g., 1918 H1N1, 1957 H2N2, and 1968 H3N2). However, it should be noted that at least in the 1957 and the 1968 pandemics, the PA segment was provided by a circulating human strain. We thus speculate that it was already adapted to human host restriction factors, such as HAX-1. The 2009 H1N1 pandemic virus has a very special reassortment history (43). While the PA segment is of avian origin, it does not contain a full-length PB1-F2 ORF. Potentially, the extended passage history in swine hosts allowed an adaptation to mammalian restriction factors. A reintroduction of PB1-F2 into this virus did not result in a replication advantage in human cells. It would be interesting in this context to test if swine HAX-1 can restrict avian virus PAs.

In our study, we demonstrated that PB1-F2 of H5N1 IAV interferes with the anti-IAV polymerase function of HAX-1 by competing with PA for HAX-1 binding. Binding of H5N1 PB1-F2 to HAX-1 would consequently free PA to perform its critical function for the viral polymerase. Our confocal IF pictures indicate that the PB1-F2 HAX-1 interaction likely takes place at the mitochondria, thus retaining HAX-1 apart from the nucleus. Obviously, it would be of great importance to study the localization of HAX-1, PB1-F2, and PA in the context of infection. Unfortunately, the lack of high-quality antibodies for the viral components (and low expression of these) prevented us from doing these experiments. Interestingly, all published functions of PB1-F2 seem to be executed largely by the C-terminal domain (44), the very same domain which interacts with HAX-1. Additional work is required to understand how the different functions proposed for PB1-F2 in viral pathology are connected.

In summary, our study provides evidence that the accessory protein PB1-F2 is required in zoonotic avian viruses, such as highly pathogenic H5N1 isolates, to overcome the restriction of the viral polymerase subunit PA by human HAX-1.

## MATERIALS AND METHODS

### Cells.

A549 cells (adenocarcinomic human alveolar basal epithelial cells; ATCC), A549-derived cells, and HeLa cells (adenocarcinomic human cervix epithelial cells; ATCC) were grown in DMEM/F-12 (Dulbecco's modified Eagle medium, nutrient mixture F-12; Gibco). HEK 293T (human embryonic kidney; ATCC), H1299 (human non-small cell lung carcinoma), and MDCK (Madin-Darby canine kidney; ATCC) cells were grown in DMEM (Gibco). Cell culture media were supplemented with 10% (vol/vol) heat-inactivated fetal bovine serum (Gibco) and 1% (vol/vol) penicillin-streptomycin (Pen-Strep). Cells were maintained at 37°C with 5% CO_2_ and 90% humidity.

### Plasmids.

pDZ plasmids contain a bidirectional expression cassette for a given influenza A virus gene segment and have been described previously (45). pCAGGS-based expression plasmids contain protein-coding sequences under the control of the chicken β-actin promoter (46). pMD2.G and psPAX2 were a gift from Didier Trono (Addgene; plasmid 12259).

pLVX-IRES-ZsGreen and pLVX-IRES-Puro were purchased from Clontech (respectively, 632187 and 632183). pSpCas9(BB)-2A-GFP (PX458) was a gift from Feng Zhang (Addgene; plasmid 48138) (47). BiFC expression plasmids for split-YFP were described previously (20). pmTurquoise2-Mito and pmTurquoise2-ER were a gift from Dorus Gadella (Addgene; plasmids 36208 and 36204, respectively) (48).

### Antibodies.

Antibodies for immunoblotting and immunofluorescence included mouse anti-actin-horseradish peroxidase (anti-actin-HRP; Abcam; ab49900), rabbit anti-HAX-1 (Abcam; ab137613), rabbit anti-influenza virus NP (Thermo Fisher; 32242), mouse monoclonal antibody against influenza A virus M1 (Bio-Rad; MCA401), mouse anti-Flag-HRP (Sigma; A8592), mouse anti-V5 (Invitrogen; R960-25), and rabbit anti-H5N1 PB1-F2 clone 9947, described previously (8).

### Oligonucleotides.

Oligonucleotides for site-directed mutagenesis were purchased from Microsynth (France) and included the following: VN/1203_C153G_fw (CACCATGGACACAGTGAACAGAACACACCAATATTC) and VN/1203_C153G_rev (GAATATTGGTGTGTTCTGTTCACTGTGTCCATGGTG), VN/1203_C222G_fw (CTGGAGCACCCCAACTGAACCCGATTGATGGACC) and VN/1203_C222G_rev (GGTCCATCAATCGGGTTCAGTTGGGGTGCTCCAG), PR/8_C153G_fw (CACCATGGATACTGTGAACAGGACACATCAG) and PR/8_C153G_rev (CTGATGTGTCCTGTTCACAGTATCCATGGTG), PR/8_C222G_fw (GGAGCACCGCAACTGAACCCGATTGATGGGC) and PR/8_C222G_rev (GCCCATCAATCGGGTTCAGTTGCGGTGCTCC), Wy/03_C153A_fw (CACCATGGACACAGTAAACAGAACACACC) and Wy/03_C153A_rev (GGTGTGTTCTGTTTACTGTGTCCATGGTG), and Wy/03_C222A_fw (GGGGCACCCCAACTAAACCCAATTGATGGAC) and Wy/03_C222A_rev (GTCCATCAATTGGGTTTAGTTGGGGTGCCCC).

### Viruses.

Recombinant viruses were produced using the eight-plasmid rescue system (45). Briefly, 35-mm dishes of subconfluent 293T cells were transfected with 0.5 μg of each pDZ plasmid using Lipofectamine 2000 transfection reagent (Thermo Fisher). Twenty-four hours posttransfection, 200 μl of 293T supernatant was used to infect 10-day-old chicken eggs. Allantoic fluids were harvest 44 h postinfection. Unique viral clones were isolated after plaque assay on MDCK cells and grown in eggs, and their genomes were fully sequenced. In this study, we used a low-pathogenicity H5N1 virus (A/Viet Nam/1203/04, Halo [18]), an H1N1 virus (A/Puerto Rico/8/1934), and a human H3N2 virus (A/Wyoming/03/2003). The PB1-F2 mutants of those viruses were generated by introduction of two stop codons (by replacing C153 with G or A and C222 with G or A) by site-directed mutagenesis using the pairs of oligonucleotides listed above. These changes do not affect the amino acid sequence of PB1. The introduction of the mutation was verified by sequencing, and its efficiency was assessed by Western blotting (WB) using polyclonal rabbit serum against PB1-F2 (8).

### Mass spectrometry.

Fifteen 10-cm dishes of 293T cells were transfected with 25 ng of pCAGGS.Flag-VN.PB1-F2 each using Lipofectamine 2000. Twenty-four hours posttransfection, cells were infected with A/Viet Nam/1203/2004 (Halo) PB1ΔF2 at a multiplicity of infection (MOI) of 1. Cells were collected 8 h postinfection by scraping, washed in phosphate-buffered saline (PBS), pelleted, and lysed in 20 ml of cold lysis buffer (0.25% [vol/vol] NP-40, 50 mM Tris-HCl [pH 7.5], 200 mM NaCl, 1 mM EDTA, protease, phosphatase, inhibitors, and *N*-ethylmaleimide [NEM]). Cells were mechanically disrupted with a Dounce homogenizer and sonicated with a Bioruptor at 4°C for five cycles (30 s on and 30 s off). Insoluble material was removed by centrifugation at 15,000 rpm for 20 min. The supernatant was precleared with nonspecific beads for 3 h at 4°C. The precleared lysate was mixed with 50 μl of beads coupled to anti-Flag (Dynabeads, M2; Sigma) and immunoprecipitation (IP) was performed for 3 h at 4°C. Beads were then washed four times with cold buffer (50 mM Tris-HCl [pH 7.5], 200 mM NaCl, 0.25% NP-40, and 1 mM EDTA) and two times with detergent-free buffer to avoid interference with mass spectrometry analysis. Beads were eluted three times with 3×Flag peptide (Sigma) in a buffer of 50 mM Tris-HCl (pH 7.5) and 200 mM NaCl. The eluted material was subjected to acetone precipitation. Resuspended proteins were digested with trypsin and then analyzed via mass spectrometry. For the identification of PB1-F2-specific interactors, we first excluded proteins precipitated from cells transfected with empty vector (see Table S2 in the supplemental material [based on area score]). The remaining proteins were filtered against the CRAPome database (49). The resulting score indicates the number of published experiments in which a target protein was pulled down with a specific antigen tag (Flag in this study). Consequently, the lower this score, the higher the chance for a specific pulldown.

### Generation of a KO cell line using CRISPR/Cas9.

Subconfluent A549, 293T, or H1299 cells in 35-mm dishes were transfected with 1 μg of plasmid pSP.Cas9wt.GFP or pSP.Cas9wt.GFP.HAX1sgRNA2.3 using 3 μl of Trans-IT LT1 (Mirus) according to the manufacturer's protocol. Seventy-two hours posttransfection, green fluorescent protein (GFP)-positive cells were sorted and seeded at 100 per 10-cm dish. Fourteen days later, colonies grown from single cells were picked and expanded. The efficiency of knockout was assessed by Western blotting. Cells generated using a guide RNA directed against HAX-1 are named A549 HAX KO and those using a random guide RNA are named A549 ctrl 2.

### Generation of overexpressing cell lines with lentivirus.

Subconfluent 293T cells were transfected at a ratio of 1:3:4 with the following plasmids: pMD2.G (vesicular stomatitis virus G protein [VSV-G]), psPAX2 (HIV gag-pol), and pLVX.HAX1.IRESpuro or pLVX.ZsGreen.IRESpuro using 2 μl/μg of DNA of Trans-IT LT1 (Mirus) according to the manufacturer's protocol. Twenty-four hours posttransfection, 293T medium was replaced with target cell medium. Target cells (A549) were seeded in a 6-well plate at subconfluent density (50%). At 48 h posttransfection, the 293T cell supernatant containing lentiviruses was harvested with a syringe and pressed slowly through a 0.44-μm sterile filter. The filtered supernatant was complemented with 8 μg/ml of Polybrene. Target cells were washed one time with PBS before addition of 2 ml of the supernatant-Polybrene mixture. Four hours after infection with lentiviruses, the 293T cell supernatant was removed from target cells and replaced with the appropriate medium. Two days postinfection, target cells were split and subjected to selection using puromycin at 2 μg/ml. The efficiency of overexpression was assessed by WB. Cells generated using a vector expressing HAX-1 are named A549 HAX OE, and those using an empty vector are named A549 ctrl 1.

### Viral growth kinetics.

Different A549 cell lines were seeded in 12-well plates at subconfluent density (80%) and infected with each virus at an MOI of 0.01 in triplicate in the presence of l-1-tosylamide-2-phenylethyl chloromethyl ketone TPCK-treated trypsin (Sigma; T1426). Supernatants were collected at the desired time postinfection. Virus titers were determined by plaque assay in MDCK cells.

### Plaque assay.

A confluent monolayer of MDCK cells was infected with 200 μl of serially diluted virus. Viruses were diluted in PBS–0.2% (wt/vol) bovine serum albumin (BSA) (Millipore; 126579). Forty-five minutes postinfection, the inoculum was removed and an agarose overlay (final concentrations: 1× minimal essential medium [MEM], 1% agarose, 100 mM l-glutamine, 2.5% sodium bicarbonate, 0.5 M HEPES, 5 mg/ml of Pen-Strep, 0.2% BSA, and 0.01% DEAE-dextran) was added. Cells were incubated at 37°C for 48 h. Then cells were fixed in formaldehyde, the overlay was removed, and the cell monolayer was stained with a solution of crystal violet.

### IP assay.

Subconfluent 293T cells (35 mm dish) were cotransfected with 1 μg of each plasmid using 2 μl/μg DNA of Trans-IT LT1 (Mirus) according to the manufacturer's protocol. Forty-eight hours posttransfection, cells were washed twice in cold PBS and then lysed on ice with 300 μl of IP lysis buffer (50 mM Tris-HCl [pH 7.5], 150 mM NaCl, 0.5 mM EDTA, 0.5% [vol/vol] NP-40) with protease inhibitors (Pierce; 88266). Samples were sonicated twice for 10 s using a microtip (output, 6 to 9 W). Insoluble material was removed by centrifugation for 20 min at 10,000 relative centrifugal force (rcf) and 4°C. Fifty microliters of the cleared lysate was mixed 1:1 with urea disruption buffer (6.25 M urea, 15% [vol/vol] β-mercaptoethanol, 4.5% [wt/vol] SDS, and 0.1% bromophenol blue) to serve as a cell lysate control, while the remaining 200 μl was processed for immunoprecipitation using anti-Flag M2 affinity gel (Sigma; A2220). Thirty microliters of agarose beads slurry was washed four times in 1 ml of cold IP lysis buffer with spin at 4°C for 5 min at 500 rcf. Beads were then resuspended in 800 μl of IP lysis buffer with protease inhibitor, and 200 μl of cell lysate was added. The immunoprecipitation was performed overnight (o/n) at 4°C under constant rotation. Then beads were washed six times in IP lysis buffer. Samples were kept on ice and tubes were changed after two washes. After the sixth wash, 70 μl of urea disruption buffer was added for 15 min. Samples were heated at 85°C for 5 min and then centrifuged at 10,000 rcf for 10 min. Samples were then used in a Western blot. When mentioned, we performed sequential IP. In short, 293T cells were transfected with only one plasmid. Twenty-four hours posttransfection, cells were lysed with 300 μl of IP lysis buffer with protease inhibitors, sonicated, and centrifuged. Fifty microliters of the cleared lysate was mixed 1:1 with urea disruption buffer. The first round of IP was performed o/n using 200 μl of samples transfected with the bait. The bead-bait complexes were washed six times and then added in 200 μl of samples transfected with the prey for a second round of IP o/n. The potential bead-bait-prey complexes were washed and disrupted in urea buffer before being processed through WB.

### Immunofluorescence (IF) assay.

Subconfluent HeLa cells, seeded on glass coverslips (24-well plate), were transfected with 250 ng of each desired plasmid using 3 μl/μg of DNA of Trans-IT LT1 (Mirus) according to the manufacturer's protocol. Forty-eight hours posttransfection, cells were washed once in PBS and then fixed in PBS–4% formaldehyde for 30 min. Then cells were washed twice and permeabilized with PBS–0.1% Triton X-100 for 15 min. The PBS-Triton was removed and cells were blocked in PBA–1% BSA for 1 h. Primary antibody directed against the protein tag was diluted at 1:100 in PBS–1% BSA and incubated for 1 h 30 min. After three washes in PBS–1% BSA, cells were incubated with a secondary antibody coupled to Alexa Fluor at 5 μg/ml for 1 h in the dark. Cells were washed twice in PBS–1% BSA and once in PBS before being mounted on a coverslip using Prolong Diamond antifade mounting medium with or without 4′,6-diamidino-2-phenylindole (DAPI; Thermo Fisher). Pictures were taken using a confocal microscope (Leica SP5).

### Bimolecular fluorescence complementation (BiFC) assay.

Subconfluent HeLa cells were transfected with 250 ng of each desired plasmid using 3 μl/μg of DNA of Trans-IT LT1 (Mirus) according to the manufacturer's protocol. Forty hours posttransfection, cells were shifted at 33°C for 4 h to stabilize YFP complexes. Pictures were taken using the EVOS cell imaging system (Thermo Fisher).

### Mini-replicon assay.

Subconfluent A549, 293T, or H1299 cells lacking HAX-1 expression were cotransfected with expression plasmids for the viral proteins VN PB2, PB1, PA, and NP as well as a plasmid carrying a firefly luciferase-based minigenome and one encoding Renilla luciferase under the control of a constitutive promoter (50). The mini-replicon system of A/Viet Nam/1203/2004 (H5N1) and A/Puerto Rico/8/1934 (H3N2) and pDZ-PA of A/Shanghai/1/2013 (H7N9) were kindly provided by P. Palese (Icahn School of Medicine at Mount Sinai, New York, NY). pCAGGS-PA of A/Brevig Mission/1/1918 was kindly provided by M. Schwemmle, University of Freiburg, Germany. When indicated, different amounts of HAX-1 and/or PB1-F2 protein-coding plasmids were added to the system. Twenty-four hours posttransfection, cells were lysed and the luciferase assay ws performed using a dual-luciferase reporter assay system (Promega).

### Apoptosis assay.

Cells were seeded in 12-well plates at subconfluent density (70%) and infected with each virus at an MOI of 5 without TPCK-treated trypsin. Twenty-four hours postinfection, cells were harvested using trypsin-EDTA and processed through staining for apoptosis markers. For this protocol, all spins were performed for 5 min at 4°C at 400 rcf and all solutions were used at 4°C unless mentioned otherwise. Cells were washed twice with 1 ml of PBS, resuspended in 100 μl of PBS with 1 μl of Zombie Red fixable viability dye (BioLegend), and incubated 30 min at 4°C in the dark. Then cells were washed twice with 1 ml of PBS–2% BSA and once with annexin V binding buffer (BioLegend) at room temperature (RT). Cells were incubated for 15 min at RT with 5 μl of annexin V (BioLegend) diluted in 100 μl of annexin V binding buffer. Then cells were washed once in annexin V buffer and once in PBS supplemented with calcium and magnesium (PBS-Ca-Mg). Cells were fixed in 200 μl of 2% formaldehyde diluted in PBS-Ca-Mg for 20 min at RT. Cells were washed twice in PBS-Ca-Mg and then resuspended in 200 μl of PBS-Ca-Mg. Samples were analyzed by flow cytometry using a Gallios 4 (Beckman Coulter) and results were analyzed with the software Kaluza Analysis 1.5a.

### Statistical analysis.

Statistical analysis was performed using GraphPad Prism 6. Statistical tests applied are indicated in each respective figure legend.

## Supplementary Material

Supplemental material
