# Supplementary material for: H5N1 Influenza A Virus PB1-F2 Relieves HAX-1-Mediated Restriction of Avian Virus Polymerase PA in Human Lung Cells
Source: J Virol. 2018 May 14;92(11):e00425-18. doi: 10.1128/JVI.00425-18 (PMC5952157; doi:10.1128/JVI.00425-18)
Supplement: Supplemental material [file supp_92_11_e00425-18__index.html]

H5N1 Influenza A Virus PB1-F2 Relieves HAX-1-Mediated Restriction of Avian Virus Polymerase PA in Human Lung Cells — Supplemental material 

# H5N1 Influenza A Virus PB1-F2 Relieves HAX-1-Mediated Restriction of Avian Virus Polymerase PA in Human Lung Cells

## Supplemental material

- Supplemental file 1 -

  Table S1 (Identification of host protein interactors of IAV PB1-F2 by mass spectrometry.)

  XLSX, 497K
- Supplemental file 2 -

  Table S2 (Host interactors from Table S1 ranked according to their abundance in a set of 411 mass spectrometry experiments.)

  XLSX, 49K
